# Glossolalalararium Pandemiconium: A Meaningfully Irreverent, Queerelously Autoethnographic Essamblage for Trying Times

**DOI:** 10.1177/1077800420960144

**Published:** 2021-09

**Authors:** Peta Murray

**Affiliations:** 1 RMIT University, Melbourne, Victoria, Australia

**Keywords:** queering, queering autoethnography, essay, bricolage, neologism, COVID-19, practice-led research, arts-based research, autoethnographic methodologies, massive and micro, prepositional thinking, meaningful irreverence

## Abstract

If there is any throughline to COVID-19, it lies within a narrative of capriciousness. To explore the paradox of the pandemic as proliferation meets obliteration, I alternate randomization with redaction within a riotous *essamblage* combining essayistic principles of “prepositional thinking” with bricolage. Neologisms meet *textracts* of responses to the 21 prompts of the Massive and Micro experiment. *Glossolalalararium Pandemiconium* comes with a helping of “lala” in a froth of babble, doubt, rage, and whimsy. It is a textual grappling with the shock and awe of the everyday, girded by a notion of “meaningful irreverence” that underpins my current research.

## Pandemiconium^1^

In the early days, I found myself lost for words. Not because I could not find words, but because the words I needed did not exist in the English I spoke, wrote, made with or through or by. As a former playwright, emerging academic and sometime essayist, I saw language as pliable matter and considered myself a wordsmith yet finding myself in need of a new lexicon for unprecedented times, I set forth to make one.

## Anthropocoronascene^2^

One Webster Pack seven days three multis one vit C one turmeric caps one zinc caps extracted from foil-backed blister pack of 10 tabs labeled by hand with black Sharpie® PERMANENT MARKER—MARQUER PERMANENT—Chisel Tip Pointe Biseautee Punta Bisalada one pack ends say Wednesday as we used to know it next must be labeled with care TH, F, SA, SU, M, TU, W, TH, F, SA my pharmacopeia horological blister pack time piece dose of the day by day, day by day things I count clockwise anticlockwise clockwisdom anticlockwisdom intuitive it’s all in the detail Moandays, Blursdays, Satalldays and every other daze one or even two pillules—is that a word, pillule, and why does it autocorrect to pillage—and how does it differ from a Circadin-MR 2mg prolonged release tablet containing melatonin from a box of 30 tablets Protect from light and Nil Repeats.

## Sundrowning^3^

I am your tawny malt, your caramel comfort, your medicinal snort at the end of a day. I am your tonic, your reward, the sandpaper on your sensitivities, buffing and blunting the splinters of over-stuffed, long, loud, hyper-stimulating meetings, this endless looking, curating yourself for virtual consumption. Soft on the tongue, and soothing to the throat, warming to the belly, and easeful on the nerves, I am a wee whiskey witch, a prelude to the descent, a clack of ice cubes in a glass, happy hour handbag, preamble to nightly news, your burnisher of day’s ends.

## Nunnify^4^

Woman One brings the blades to Woman Two’s head and begins to shear her hair. A slow mo-mow. We see the clippers push against the hair and shave it from the head. The first signs of pale *scalpture.*^5^ A lunar landscape. Woman One stops. Woman Two rubs her hand over her head. Woman One resumes the clip. Woman One circles Woman Two, north, south, east, west. Woman Two’s smile grows broader and broader. Now shorn of all hair she casts off the garment and shakes her body like a wet dog. Hair suspended in air. Freeze frame.

## Salutory Confinement^6^

The now or neverness of things powered by the unfathomable enormity of it. The impossibility of pitching one’s will, or stamping one’s foot, or making any difference to anything. Not a yielding or an acceptance but a friction arising in that gap between what might be desperation and a kind of collapsed surrender. There’s a bristling resistance, and it’s that energy, if anything, that can move things. A refusal to buckle, yet with a tip o’ the hat, a small obeisance, a nod to greater forces at work as one tries to find grace in the buffeting chaos of it all.

## Hiberactive^7^

You’re asking me about nature today, just when I have started having a soft spot for machines. Nature, in the time of COVID, I envy you. I envy you, bird and you tree, you, kangaroo and you, my small brown dog starting your evening barkathon as the night comes down, or in, or over like a blanket. Your contempt for me, for us, never shown, you go on flying, landing, bending, shading, molting, breeding, barking.

## Unschoolfulness^8^

My iPhone. And that old-fashioned red lamp with the bendy-flexy stand, crimson shade. Is the fire box–wood heater–a machine or a beast that must be fed twigs and kindling and balls of newspaper and log after log in return for a steady generosity of warmth? The vacuum cleaner in that cupboard. The kitchen is machine city: the dishwasher, the coffee machine, and the kettle, we’re surrounded and if they rise up against us, refrigerator, stove and cooktop, toaster, and NutriBullet with its killer blades. The downstairs bedroom, lamps, are we breeding lamps? The inevitable iPad. Finally, my ersatz covidoffice. Lamps and lamps and a Vintage Printer. Paper jams so rare in the old beast which keeps on printing, years after its planned obsolescence. How little respect I had for it, or for this cohort of the cogged and the calibrated, the charged and the battery-operated, the bladed and the blunt. Till now. Is it weird to bow down before you, Brother MFC-5860CN? And why have I not made time to learn your name?

## Travellater^9^

Cartographies of New Nowland. You are here. They are there. Outside, the Provinces of Postponement, Cancelation, Deferral, and Delay. They are on the outskirts of the outskirts of the Outpost of Offloading. Not far from Concatenation. Quite close to Tilfurthernotice. Within coo-ee of Lock, Stock, and Barrel.

## Rehumanising^10^

Rufus consoled, for a while, but now I find him far too noisy. The nightly news anthem, and the play-in before Coronacast is about all that I can manage. Tolerate. Muster. Take your pick. In the words, though, there’s my music. In the contortions and distortions of language and in the nightly spillage, the overflow, the allophony. Here I find pleasure and reason, in the mellifluousness of this sound beside that, this consonant beside that diphthong, the jar and the jolt of that plosive beside that anapaest. How much do I love each of these words, the way sound is arrayed in them and what has it to do with COVID, nothing, with pandemic, nothing, with climate change, nothing, with the new world disorder, nothing.

## Fomophobia^11^

If you listen you can hear the Sea of Chaos. You are just a shred of Flotsam or Jetsam—it matters not which—bobbing about in its immensity. It cares nothing for you. You are a mere bubble in the bubblesomeness of its foam, a mere speck in its phosphorescence, a mere smear on the surface of its slick and sheen. Be tossed. Try to enjoy it. Life is short and you are a grain of salt in the salad dressing and nothing more. It’s still now. The sun is low. There is a dance in progress between loss and joy, joy and loss. They break the water’s surface and glisten a moment, above, like flying fish.

## Cloisterfuck^12^

A new mask, this prototype adds oxygen, it appears, making for *a cleaner breathing experience* and knowing one day I may be able to afford better quality air than you can, it all seems so wrong. But this, this is a mask for good.

## Zoombie^13^

In nearby Clocktown, stillness. No alarms. No imperatives. No urgencies. The diurnal churn continues and you can catch up on some sleep and no-one will ever know. You no longer understand what any of this means, and when you do sleep, your dream districts are all red light and you stagger from nightmare to reverie, with a drink in your hand, in an unwashed gown. You love these words now, how they slump on each others’ shoulders, and you just want to leave tiny stains and traces on things with inky hands before you cast yourself into tomorrow, and tomorrow, and tomorrow.

## Pellage^14^

Men. White men. Old White men. The future is only visible if they are somehow persuaded to loosen their grip on the world. Yesterday, on a bench outside the church was a tomato sauce-stained chip box, and on the ground plastic forks and serviettes and empty bottles of a premixed bourbon drink. Note the performance of not-caring this diorama portrays. A mise-en-scène in the remains of a snack stop by men who have enacted for the benefit of one another their contempt for care. Who have performed their scorn for the softness of care for each other, for earth, land, country. Is this what care-free means and if so how can we slice through this thick-skinnedness and make it pulpy enough to allow feeling to course where it has been locked inside muscle and meat?

## Iso-Trope^15^

The sunset is yours too. The stars. The cold air. The dirt, the mulch, the leaf litter, the moss, the marvelous moss. I love you moss. Is nature queer? Let’s say so. Let’s see nature bend and keep bending, let’s see her limber enough to slip right underneath us humankinds and limbo dance out the other side in forms we can’t even dream of.

## Ronageist^16^

In America, rampaging looters, fires, chaos. A man lost his life under the knee of another. A man murdered a woman, a child, a man massacred, a man stormed, a man raged, a man mowed down helpless others. Street warfare, race warfare, police brutality, unfettered power of rich White men to not care, and to signal to each other that they care even less, while amassing more than they will ever need.^17^

## Legumebriousness^18^

And me, and my embodied self, and my edges and the boundaries between self and planet to be blurred and questioned. A brittle porosity, my shell, my need to move in and out of it, its luster, its chips, cracks and fissures, where the light gets in, or out, if I am emitting. Which I sometimes do. My verbal emissions. My neologologic.

## Cabination^19^

Unable to pick up a pen and put it to paper I attempt to catch it here, the music of lock down. First, in birdsong. Where I have been living it is birdsong that announces the day, punctuates the day, declares the day over. Birds are the town criers of Anglesea, and as I write that sentence I wonder if anyone ever had cause to write those words, in that order before, or will again.

## Ground-Dogging^20^

There is something so compelling here, lodged in the utter impossibility of using mere words to speak to this. I want to write: ib uyg nd kin kjhuvhmn nuhuenvciurjni evniovej. I want to write xkjck c8weuyb wee;owe;qoweuybvo. I want to speak plant and dog and cloud and rain and rainbow. I want to speak bird, and not this bird but that bird. I want to speak Border Terrier, with a wee Scot’s brogue, so I can commune with my dog, and I want to speak lamppost. I want to speak daphne and seedpod. I want to speak peppercorn tree and grevillea. I want to speak grass.

## Youthage^21^

Loretta and I went for three walks today. It was icy, first up. Decaying leaves were slippery under my feet and there was possum scat and Loretta tried to eat it. I ate all the wrong things too for breakfast. Tropical fruits. And bagels. Where do I think I am? Outside, the peppercorn trees loom larger than all others. The torsions and extensions of their balletomane bodies, the elevation. These are trees with tone and heft. I cannot hear them speak, much as I want to. But I smell them. I see them brush their leaves against the bluestone in a murmuration and admire them deeply, in their humility and grace. Respect.

## Lullabide^22^

Second walk is around the block. Decaying ginkgo leaves, their stinking fruit. Odd succulents around trees, where humans have decided to green their patch of street, now *greening* is a verb. Third walk, longer, but by now, my receptors are blunted by a long work day on a day one does not usually work. How to hear *into*? How to listen *through*?

## Jigsorcery^23^

The dramaturgy of the quotidian prepositional, the deep soundings of the animate, the pulse of life and liveness, living and lithe. The flicker. The frisson. If only. If only. Guwqyhwekn k. Yes, of course. Jeshiuwehlrjkeoi. I can hear you. Xb gouiheeopwiu. Do you hear me? Od unvijrkqhvghwgfevv.,[pd i3urn. Speak to me. I’m listening. I’m listening. I’m here.

## After-Words

The compositional complicity of subject-world is, for me, the first question of autoethnography. It is a way of calling up the textures and densities of worlds of all kinds formed out of this and that—identities, situations, scenes, sensory conditions, bodies, meanings, weights, rhythms, absence. A way of sidling up to the interesting or unbearable atmospheres and events of those worlds. A hinge onto a moment of some world’s legibility. (Stewart, 2013, p. 667)

In March 2020, I began publishing a neologism a day at a personal blogsite called Mmmmy Corona. When the Massive_Microscopic_Sensemaking Project (hereafter MMSP)^24^ began I found new apertures through which to splice the *micro-w/rites* of my *be-musings* onto a communal canvas. As Ross Gibson writes,

After all, it is from these quotidian montages that epiphanies sometimes spark. And through these tiny fissures in commonsense, so long as you can believe that a logic other than chance or chaos subtends them, you might eventually register some gleams of new understanding about the forces that jostle people and nature in space throughout time. (Gibson, 2015, p. 34)

My epiphany of 2020 is that if there are any throughlines to COVID-19, they lie within narratives of capriciousness. This word denotes both sudden unaccountable changes of behavior or mood or else changes arising, unpredictably, according to no discernible rules. To reflect this dynamism, I adopt capriciously *queerelous* methods, reducing 10,000 words generated in reply to 21 prompts of the MMSP into 21 *textracts*, under 21^25^ neologisms, chosen and distributed at random^26^ as sub-headings. The resultant *essamblage*,^27^ with its virulent twin-engines, merges genre-bending notions of the essayistic (Singer & Walker, 2013) with ideas of bricolage as made through working with what lies at hand.^28^ In so doing, it illustrates how rhetorical instruments may be used to disrupt *and* order, clarify *and* subvert (Murray, 2017, p. 254).

To underwrite some of the disorientations and precarities of this cultural moment, I also pay *homomage*^29^ to controversial rhetorical theorist, the late Mary Daly, whose *cunnilinguistic* texts were formative for many feminists of my generation. I remain influenced, as other queer rhetoricians are, by how Daly “worked with and in and through [words]; the way she talked about their power, or rather a woman’s/womyn’s power and ability to challenge the patriarchy through the conscious manipulation and transformation of them” (Hawkins, 2018, p. 191). Such *trans-for-motions*, I find, embolden me to work the hyphens, as Michelle Fine would say (Fine, 1994) moving through them toward a beyond where I find myself making a MIQAE,^30^ a new method for queering autoethnography, that is, I propose, a fitting response to “the erasures and violences that punctuate our everyday” (Holman Jones & Harris, 2019, p. 119).

Making a MIQAE deploys ideas from creative writing scholarship such as prepositional thinking whereby “[t]he rub between edges, the crossing of thresholds, insides and outsides, outsides with insides . . . creates new neural pathways, allows for slantness, oblique and not so oblique resistances, push-through, and importantly, processual thinking” (Rendle-Short, 2020, p. 7), *alongside* material practices that perform “the poetics of the disjunctive and the awkward combinations of fitting and not fitting . . .” (Walker, 2014, p. 217). For if “queerness, like activism, is a way of being, a mode, perspective, an enactment of citizenship and a not-yet” (Holman Jones & Harris, 2019, p. 65), then *qae* (queerelous autoethnographic essamblage) is a form of becoming, anti-literary, *proprioperformative*, and prepositionally promiscuous such that “that which is lodged alongside, between and around, and under the words you read here, is where its meaning resides” (Murray, 2017, p. 279).

I offer this riotous *essamblage* as one more *qae* to that other door held resolutely ajar by Holman Jones and Harris (2019), having been prised open by queer and feminist thinkers before them, when they exhort those of us who would explore the complexities of critical sense-making to commit to “an evolution in which the story as enactment becomes something more than a recording of past events” (p. 64.) It performs my commitment to w/rites of “meaningful irreverence” that give shape to my grapplings inside The Great Pause. At the same time, it illustrates potencies of artful creative practice to expose meanings inside the mayhem, rage, fear, and uncertainty of the pandemic and beyond into the massive and long-awaited sociopolitical shockwaves that now ensue.

## Notes

1. Not so much a word as a place we seem to be inhabiting suddenly, and without much warning. Murray (2020l).

2. The Anthropocoronascene can be seen through the screen of the Anthropocoronacene. It sits within a kaleidoscope of queer subcultures and is best viewed through the screen of “scenes”—Murray (2020a).

3. The urge to drink, long before the sun is past the yard-arm, with wine o’clock still many hours away—Murray (2020s).

4. To so simplify one’s life as to realize one can live well with fewer choices, fewer decisions, fewer interactions with the world—Murray (2020k).

5. The satisfaction and sensory pleasure to be found in new forms, especially off the top off one’s head—Murray (2020r).

6. Remaining contained and physically distanced for our own well-being—Murray (2020q).

7. Being more active under lockdown that one ever would be in “normal” daily life—Murray (2020f).

8. Feelings of ineptitude that may accompany any attempt to teach oneself, or others, via the platforms of the online learning world—Murray (2020u).

9. Travel undertaken without luggage or tickets, wherein, through photos or memories, souvenirs, or reverie, you are transported all the same—Murray (2020t).

10. Turning back into a human being under the influence of time and space—Murray (2020o).

11. The fear of missing out on the feeling of missing out—Murray (2020d).

12. The intrusion of a possibly virus-bearing entity into a sanitary sanctuary—Murray (2020n).

13. The state of almost catatonic exhaustion reached by the end of a 5-day work week in which one has been tele-conferencing for at least 2 hr of each day—Murray (2020w).

14. Destructive and damaging acts taking place under cover of COVID (or Commerce, the Commonwealth or Catholicism), while the people’s attention is turned elsewhere and they are COVIDazzled—Murray (2020m).

15. Compound word formed by adding *iso-* (Greek for “equal”) to -*trope* (Greek for turn or way). An iso-trope occurs wherever the word *iso-* is used in a significant, metaphorical, or recurring way to speak of #isolife under COVID-19 lockdown.—Murray (2020g).

16. The defining spirit or mood of a period of history as modeled by the behavior of the most virulent virus of its times—Murray (2020p).

17. It was the matter of masculinity rather than the race issue that drew me and my white feminist eye here. I acknowledge this is not how BIPOC communities may interpret these events in America. I admit too that while naming America here gives the sentence more punch than calling upon the United States, it also erases the rest of the Americas both North and South of this Ronageist. Ah, language, you wily wench.

18. Feelings of lethargy induced when one opens one’s pantry cupboard to the stockpile of chickpeas, lentils, and kidney beans one has yet to consume under lockdown—Murray (2020i).

19. Cabination: A chemical reaction that may occur within a confined space when *cabinfever* meets *cabinfervour*, giving rise to a burst of resplendent creativity—Murray (2020b).

20. The capacity to repeat a routine without ever seeming to tire of it—Murray (2020e).

21. Months of life lost wondering when the Anthropocoronacene will end—Murray (2020v).

22. A temporary interval of quiet, or lack of activity, a pause in which we may settle into accordance, even *detente*, with the way of the world, just as it is, in this fleeting moment. And then it is gone—Murray (2020j).

23. The capacity of a jigsaw to hold you under its spell until all measures of time, place, meaning, or purpose are lost—Murray (2020h).

24. See Markham and Harris (2020).

25. The only significance of these numbers is to mirror of the 21-day cycle of the MMSP experiment conducted by Harris and Markham.

26. See www.randomlists.com.

27. A playful construction made by combining essayistic text with notions of the multidimensional assemblage including found objects—Murray (2020c).

28. Or beyond reach of the hand? All the more valuable as I essamble this final draft with a trifracture of broken finger, wrist, and ankle.

29. A word made on the run, in queer homage.

30. Making a MIQAE (pronounced mickey) is a queer variation of taking the mickey, an idiomatic expression used throughout the Commonwealth to denote making fun of someone or something. Making a MIQAE flips the passivity of taking into the proactivism of making.

## References

[bibr1-1077800420960144] Fine M. (1994). Working the hyphens: Reinventing the self and other in qualitative research. In Denzin N. K. Lincoln Y. S. (Eds.), Handbook of qualitative research (pp. 70–82). SAGE.

[bibr2-1077800420960144] Gibson R. (2015). Memoryscopes: Remnants forensics aesthetics. UWAP Scholarly.

[bibr3-1077800420960144] Hawkins A. (2018). Daly masturbations of/and/by our dear Lorde. PRE/TEXT, 24(1–4), 189–206.

[bibr4-1077800420960144] Holman Jones S. Harris A. M. (2019). Queering autoethnography. Routledge.

[bibr5-1077800420960144] Markham A. Harris A. (2020). Massive and microscopic sense-making in the time of COVID. Call for expressions of interest. https://futuremaking.space/call-for-participation/

[bibr6-1077800420960144] Murray P. (2017). Essayesque dismemoir: W/rites of elder-flowering [Unpublished doctoral dissertation]. RMIT University.

[bibr7-1077800420960144] Murray P. (2020a, July 30). Neologism of the day: Anthropocoronascene. WordPress. https://mmmmycorona.wordpress.com/2020/07/08/30-july-2020/

[bibr8-1077800420960144] Murray P. (2020b, May 5). Neologism of the day: Cabination. WordPress. https://mmmmycorona.wordpress.com/2020/05/05/5-may-2020/

[bibr9-1077800420960144] Murray P. (2020c, June 19). Neologism of the day: Essaimblage. WordPress. https://mmmmycorona.wordpress.com/2020/06/19/19-june2020/

[bibr10-1077800420960144] Murray P. (2020d, April 6). Neologism of the day: Fomophobia. WordPress. https://mmmmycorona.wordpress.com/2020/04/06/6-april-2020/

[bibr11-1077800420960144] Murray P. (2020e, April 3). Neologism of the day: Ground-dogging. WordPress. https://mmmmycorona.wordpress.com/2020/04/03/3-april-2020/

[bibr12-1077800420960144] Murray P. (2020f, April 18). Neologism of the day: Hiberactive. WordPress. https://mmmmycorona.wordpress.com/2020/04/18/18-april-2020/

[bibr13-1077800420960144] Murray P. (2020g, April 28). Neologism of the day: Iso-trope. WordPress. https://mmmmycorona.wordpress.com/2020/04/28/28-april-2020/

[bibr14-1077800420960144] Murray P. (2020h, April 16). Neologism of the day: Jigsorcery. WordPress. https://mmmmycorona.wordpress.com/2020/04/16/16-april-2020/

[bibr15-1077800420960144] Murray P. (2020i, July 12). Neologism of the day: Legumebriousness. WordPress. https://mmmmycorona.wordpress.com/2020/06/12/12-june-2020/

[bibr16-1077800420960144] Murray P. (2020j, April 16). Neologism of the day: Lullabide. WordPress. https://mmmmycorona.wordpress.com/2020/07/21/21-july-2020/

[bibr17-1077800420960144] Murray P. (2020k, April 21). Neologism of the day: Nunnify. WordPress. https://mmmmycorona.wordpress.com/2020/04/21/21-april-2020/

[bibr18-1077800420960144] Murray P. (2020l, March 23). Neologism of the day: Pandemiconium. WordPress. https://mmmmycorona.wordpress.com/2020/03/23/monday-23-march-2020/

[bibr19-1077800420960144] Murray P. (2020m, April 22). Neologism of the day: Pellage. WordPress. https://mmmmycorona.wordpress.com/2020/04/22/22-april-2020/

[bibr20-1077800420960144] Murray P. (2020n, March 25). Neologism of the day: Pre-amble. WordPress. https://mmmmycorona.wordpress.com/2020/03/25/25-march-2020/

[bibr21-1077800420960144] Murray P. (2020o, March 26). Neologism of the day: Rehumanising. WordPress. https://mmmmycorona.wordpress.com/2020/03/26/26-march-2020/

[bibr22-1077800420960144] Murray P (2020p, July 20). Neologism of the day: Ronageist. WordPress. https://mmmmycorona.wordpress.com/2020/07/20/july-20-2020/

[bibr23-1077800420960144] Murray P. (2020q, July 30). Neologism of the day: Salutary confinement. WordPress. https://mmmmycorona.wordpress.com/2020/07/09/9-july-2020-2/

[bibr24-1077800420960144] Murray P. (2020r, April 20). Neologism of the day: Scalpture. WordPress. https://mmmmycorona.wordpress.com/2020/04/20/20-april-2020/

[bibr25-1077800420960144] Murray P. (2020s, March 31). Neologism of the day: Sundrowning. WordPress. https://mmmmycorona.wordpress.com/2020/03/31/31-march-2020/

[bibr26-1077800420960144] Murray P. (2020t, July 15). Neologism of the day: Travellater. WordPress. https://mmmmycorona.wordpress.com/2020/07/15/15-july-2020/

[bibr27-1077800420960144] Murray P. (2020u, July 13). Neologism of the day: Unschoolfulness. WordPress. https://mmmmycorona.wordpress.com/2020/07/13/13-july-2020/

[bibr28-1077800420960144] Murray P. (2020v, June 11). Neologism of the day: Youthage. WordPress. https://mmmmycorona.wordpress.com/2020/06/11/11-june-2020/

[bibr29-1077800420960144] Murray P. (2020w, April 24). Neologism of the day: Zoombie. WordPress. https://mmmmycorona.wordpress.com/2020/04/24/24-april-2020/

[bibr30-1077800420960144] Rendle-Short F. (2020). Preposition as method: Creative writing research and prepositional thinking, methodologically speaking. New Writing. Published 17 March, 2020. pp. 1–13. 10.1080/14790726.2020.1726967

[bibr31-1077800420960144] Singer M. Walker N. (Eds.). (2013). Bending genre: Essays on creative nonfiction. Bloomsbury Academic & Professional.

[bibr32-1077800420960144] Stewart K. (2013). An Autoethnography of what happens. In Holman Jones S. Adams T. Ellis C. (Eds.), Handbook of Autoethnography (pp. 659–668). Routledge.

[bibr33-1077800420960144] Walker N. (2014). Permutation and randomness in the action score generator. Journal of Writing in Creative Practice, 7(1), 213–221. 10.1386/jwcp.7.1.213_1

